# Effects of Dietary Marine Sulfated Polysaccharides Derived From Macroalgae on Intestinal Health of Nursery Pigs

**DOI:** 10.1111/asj.70158

**Published:** 2026-02-15

**Authors:** Yi‐Chi Cheng, Yesid R. Garavito‐Duarte, Maria Garcia Suarez, Maria A. Rodríguez, Sung Woo Kim

**Affiliations:** ^1^ Department of Animal Science North Carolina State University Raleigh North Carolina USA; ^2^ Olmix Brehan France

**Keywords:** anti‐inflammatory, anti‐oxidative, intestinal health, macroalgae sulfated polysaccharide, nursery pigs

## Abstract

Post‐weaning stress in pigs leads to intestinal inflammation and oxidative stress. Macroalgae has gained attention with anti‐inflammatory and anti‐oxidative properties to mitigate these negative impacts. This study aimed to evaluate the efficacy of sulfated polysaccharides extracted from marine macroalgae (*Ulva* spp. and *Solieria chordalis* ) promoting intestinal health and growth performance in nursery pigs. Twenty‐four nursery pigs at 21 days of age, with initial body weight (BW) of 6.5 ± 0.2 kg, were allotted to dietary treatments in a randomized complete block design, with sex and BW as blocks. Pigs were assigned to either a control diet or a diet supplemented with 0.2% mixed macroalgae. Growth performance was recorded by phase. At 56 days of age (day 35 of the study), pigs were euthanized to collect jejunal tissues and mucosa to evaluate intestinal health. Supplementation with 0.2% mixed macroalgae decreased (*p* < 0.05) TNF‐α, tended to decrease (*p =* 0.064) IL‐8, and decreased (*p* < 0.05) protein carbonyl in jejunal mucosa, indicating reduced intestinal inflammation and oxidative stress, suggesting a potential protective effect of macroalgae in jejunum. In conclusion, dietary inclusion of 0.2% mixed macroalgae may serve as a functional strategy to support intestinal health in nursery pigs under inflammatory stress.

## 1 Introduction

Post‐weaning stress is a critical challenge in pig production, as nursery pigs face multiple stressors, including separation from the sow and littermates, adapting to a new environment, and transitioning from a milk‐based diet to solid feed (Campbell et al. 2013; Kim and Duarte 2021). These stressors often lead to decreased feed intake, intestinal dysbiosis, and increased susceptibility to enterotoxigenic pathogens such as *Escherichia coli* infection, the primary cause of post‐weaning diarrhea (Duarte, Stahl, and Kim 2023; Jang et al. 2023; Rhouma et al. 2017). Post‐weaning diarrhea commonly occurs within the first week after weaning and is associated with intestinal inflammation, including elevated pro‐inflammatory cytokines, oxidative stress, altered immunoglobulin levels, and compromised intestinal morphology. These mechanistic indicators are widely assessed in nutritional intervention studies (Duarte, Garavito‐Duarte, and Kim 2023; Gormley et al. 2024; Garavito‐Duarte et al. 2025), as they underline the clinical outcomes of watery diarrhea and mortality rates ranging from 1.5% to 25.0% (Duarte, Garavito‐Duarte, and Kim 2023).

Antibiotics have long been used in nursery pig diets to mitigate the negative effects of weaning stress on intestinal health and growth performance (Cromwell 2002; Sung et al. 2025). However, concerns over antimicrobial resistance have led many countries to phase out the use of antibiotic growth promoters in animal feeds (Centner 2016; Hu and Cowling 2020; Tiseo et al. 2020). Similarly, the use of high‐dose zinc oxide is restricted due to environmental concerns (Broom et al. 2006; Pejsak et al. 2023). Effective alternatives are needed to support intestinal health and performance without the risks of antibiotics and high mineral doses.

Macroalgae, commonly known as seaweed, have gained attention for their anti‐microbial, anti‐inflammatory, and anti‐oxidative effects (Czech et al. 2024; Rocha et al. 2022). They are classified based on pigmentation: brown (Phaeophyta), red (Rhodophyta), and green (Chlorophyta) (Kim 2021). As a natural alternative, macroalgae have shown potential to enhance intestinal development and nutrient utilization in pigs (Choi et al. 2016; Wan et al. 2018). The chemical composition of macroalgae varies by species, season, habitat, and environmental conditions (Kim 2021; Øverland et al. 2019). Sulfated polysaccharides from green macroalgae modulate the transcription of immune mediators involved in defense mechanisms within the innate and the adaptive immune responses (Berri et al. 2017). In vivo, recent studies further confirmed the immunomodulating properties of green macroalgae sulfated polysaccharides, namely, by improving the defense activities of monocytes and heterophils (Guriec et al. 2018), and by favoring the transfer of maternal immunity (Bussy et al. 2019). The dietary supplementation with sulfated polysaccharides extracted from marine macroalgae (*Ulva* spp., and *Solieria chordalis* ) improved growth performance and reduced intestinal lesion scores by reducing inflammatory responses in broilers in a necrotic enteritis challenge (Blue et al. 2024). Additionally, the combination of red and green algal extract enhanced mRNA abundance of tight junction proteins, which may contribute to improved intestinal integrity.

The complexity and reactivity of seaweed polysaccharides arise from their diverse and sometimes rare sugar units, such as uronic acids, xylose, and rhamnose (Blue et al. 2024). Sulfated polysaccharides from green, red, and brown seaweeds, such as ulvan, agar, carrageenan, and fucoidan, exhibit diverse molecular structures due to variations in glycosidic linkages and the presence of sulfate groups. These structural features, particularly sulfation patterns, are key determinants of their biological activity and functional properties (Akter et al. 2024; El‐Beltagi et al. 2022). Macroalgae polysaccharides, particularly sulfated polysaccharides like ulvan and carrageenan, have anti‐inflammatory and anti‐oxidative effects (Bahar et al. 2012; Bussy et al. 2019). These bioactive compounds support anti‐inflammatory pathways in intestinal immune cells (Chakraborty et al. 2023; McCauley et al. 2015; Ye et al. 2020) by inhibiting signaling cascades such as nuclear factor‐κB (NF‐κB), mitogen‐activated protein kinase (MAPK), and Janus kinase (Corino et al. 2021; Stephanie et al. 2010; Tabarsa et al. 2018). They enhance anti‐oxidative response by increasing redox enzyme activity such as superoxide dismutase, catalase, and glutathione peroxidase, which play essential roles in neutralizing reactive oxygen species (ROS) and reducing lipid peroxidation and protein oxidation (Azizi et al. 2023). Preventing oxidative damage to cellular components, including lipid membranes, proteins, and DNA (Azizi et al. 2023; Czech et al. 2024; Monroy‐García et al. 2025). Beyond their anti‐inflammatory and anti‐oxidative effects, certain algal polysaccharides such as ulvan may also contribute to intestinal health by modulating the intestinal microbiota (Pratap et al. 2022; Liang et al. 2024).

Given the background, it is hypothesized that supplementing sulfated polysaccharides extracted from marine macroalgae (*Ulva* spp. and *Solieria chordalis* ) can reduce jejunal inflammation and oxidative stress, consequently improving intestinal morphology and growth performance in nursery pigs. Therefore, this study evaluated the effects of this dietary macroalgae sulfated polysaccharide on intestinal health of nursery pigs.

## 2 Materials and Methods

The experimental protocol was approved by the Institutional Animal Care and Use Committee of North Carolina State University (Raleigh, NC). The mixed macroalgae sulfated polysaccharides used in this study Algimun (Algoguard; Olmix S. A, Bréhan, France) are a commercial product based on the combination of two bioactive macroalgae extracts: MSPBARRIER (Olmix S. A, Bréhan, France), a red algae extract ( *Solieria chordalis* ), rich in iota‐carrageenan with a content of 20% to 30% of 3,6‐anhydrogalactose, and MSPIMMUNITY (Olmix S. A, Bréhan, France), a green algae extract (*Ulva* spp) rich in ulvans.

### 2.1 Animals, Experimental Design, and Experimental Diets

Twenty‐four nursery pigs (PIC 337 × Camborough 22; 12 barrows and 12 gilts), weaned at 21 days of age, with an initial body weight (BW) of 6.5 ± 0.2 kg, were allotted to dietary treatments in a randomized complete block design, with sex and BW as blocks. Pigs were housed individually in pens with free access to feed and water. Pigs were assigned to one of two dietary treatments: a control diet or a diet supplemented with 0.2% mixed macroalgae sulfated polysaccharides. The inclusion rate of 0.2% mixed macroalgae sulfated polysaccharides was selected based on previous studies in poultry and fish using the same combination of two bioactive macroalgae extracts (Tharaka et al. 2020; Blue et al. 2024). The experimental diets were formulated to meet or exceed the nutrient requirements based on NRC (2012) for 35 days based on three phases (phase 1: 7 days; phase 2: 14 days; and phase 3: 14 days). The composition of basal diets is shown in Table 1. Body weight and feed intake were measured at the end of each phase to calculate average daily gain (ADG), average daily feed intake (ADFI), and gain to feed (G:F) ratio.

**TABLE 1:** Composition of diets in each phase (as‐fed basis).

	Phase 1	Phase 2	Phase 3
Feedstuff, %			
Corn grain	27.5	40.0	65.4
Whey permeate	24.0	15.0	—
Soybean meal	18.0	20.0	26.0
Cookie meal	10.0	10.0	—
Poultry meal	10.0	8.0	5.0
Fish meal	4.00	2.00	—
Blood plasma	4.00	2.00	—
Poultry fat	1.00	1.00	1.00
L‐Lys HCl	0.46	0.48	0.42
L‐Met	0.23	0.20	0.14
L‐Thr	0.14	0.14	0.12
L‐Trp	0.01	—	—
Dicalcium phosphate	—	0.15	0.80
Limestone	0.30	0.65	0.77
Vitamin premix^a^	0.03	0.03	0.03
Mineral premix^b^	0.15	0.15	0.15
Salt	0.22	0.22	0.22
Calculated composition			
Dry matter, %	91.2	90.6	89.5
ME, kcal/kg	3446	3427	3370
Crude protein, %	25.3	22.9	21.6
SID Lys, %	1.50	1.35	1.23
SID Met + Cys, %	0.82	0.74	0.68
SID Thr, %	0.88	0.79	0.73
SID Trp, %	0.25	0.22	0.21
Ca, %	0.85	0.81	0.71
STTD P, %	0.51	0.40	0.33
Analyzed composition, %			
Dry matter	90.9	88.6	86.9
Crude protein	24.9	23.2	19.5
Ether extract	4.76	4.70	4.11

Abbreviations: ME, metabolizable energy; SID, standardized ileal digestibility; STTD P, standardized total tract digestible phosphorus.

^a^ The vitamin premix provided the following per kilogram of complete diet: 6613.8 IU of vitamin A acetate, 992.0 IU of vitamin D_3_, 19.8 IU of vitamin E, 2.65 mg of vitamin K, 0.03 mg of vitamin B_12_, 4.63 mg of riboflavin, 18.52 mg of D‐pantothenic acid, 26.45 mg of niacin, and 0.07 biotin.

^b^ The trace mineral premix provides the following per kilogram of complete diet: 33.0 mg of Mn as manganous oxide, 109.5 mg of Fe as ferrous sulfate, 109.5 mg of Zn as zinc sulfate, 16.5 mg of Cu as copper sulfate, 0.3 mg of I as ethylenediamine dihydroiodide, and 0.3 mg of Se as sodium selenite.

### 2.2 Collection and Extraction of Sulphated Polysaccharides

Marine sulfated polysaccharides were derived from green and red macroalgae harvested from natural coastal accumulations at multiple sites in Brittany, France. Green algae were obtained from Plestin‐les‐Grèves, Guissény, and Saint‐Brieuc, whereas red algae were collected from Saint‐Hilaire‐de‐Riez and the Rhuys Peninsula. After harvesting, the algae were rinsed with fresh water, drained, frozen, and later processed by the Olmix Group (Bréhan, France). Processing included thawing, mechanical grinding, and separation of liquid and solid fractions, followed by concentration of the liquid phase to obtain an extract enriched in sulfated polysaccharides (Patent No. FR 61909).

### 2.3 Analytical Methods of Sulfated Polysaccharides

The linked sulfate content, reflecting the amount of sulfated polysaccharides, was determined for the two main families present in the product, ulvans and iota‐carrageenans. The procedure consisted of 10 g of dry algal juice solubilized in 85 mL of demineralized water and measuring the solution's conductivity. Sequential ultrafiltration using a 1‐kDa membrane was performed until the conductivity reached 0.2 mS/cm, yielding a final volume of 40 mL. The retentate was then lyophilized, and the mass yield recorded. Total sulfur content was determined using a CNS elemental analyzer, and linked sulfate was calculated under the assumption that all residual sulfur after ultrafiltration was present as sulfate bound to the polysaccharides.

### 2.4 Sample Collection

On day 35 of the study, all pigs were euthanized via exsanguination after incapacitation by a captive bolt gun to the head. After euthanasia, the digestive tract was removed for sample collection. Samples from mid‐jejunum, 3‐m distal to the pyloric‐duodenal junction, were removed and rinsed with a sterile saline solution (0.9%) to clear the sample of digesta content. A section of the mid‐jejunum was opened longitudinally, and the mucosal layer was gently scraped using a glass microscope slide. The collected mucosal tissue was then placed into 2‐mL Eppendorf tubes, rapidly frozen in liquid nitrogen, and stored at −80°C for later analysis of immune parameters, including inflammatory cytokines and immunoglobulins, as well as oxidative stress markers. A segment of the mid‐jejunum was removed, rinsed with a sterile saline solution, and placed in a 50‐mL Falcon tube containing 10% buffered formaldehyde, then stored for further evaluation of intestinal morphology, including villus height (VH), crypt depth (CD), and crypt proliferation rate.

### 2.5 Immune Status and Oxidative Stress Markers in the Jejunal Mucosa

Samples of the scraped mucosa from mid‐jejunum were weighed (1 g) and suspended in 1 mL of phosphate‐buffered saline (PBS). Mucosa samples were homogenized (Tissuemiser, Thermo Fisher Scientific) for 30 s on ice, withdrawn into new 2‐mL microcentrifuge tubes, and centrifuged at 14,000 × *g* for 15 min (MiniSpin, Eppendorf; Hamburg, Germany), following the methodology described by Holanda and Kim (2021). All supernatants were collected and stored at −80°C for further analysis.

The concentrations of total protein, interleukin‐6 (IL‐6), interleukin‐8 (IL‐8), tumor necrosis factor‐alpha (TNF‐α), immunoglobulin A (IgA), immunoglobulin G (IgG), malondialdehyde (MDA), and protein carbonyl were measured using commercial kits, following the operation manuals provided with each respective kit. The optical density (OD) value for each assay was obtained using a plate reader (Synergy HT, BioTek Instruments; Winooski, VT) and compatible software (Gen5 Data Analysis Software, BioTek Instruments). To calculate the concentrations of each respective product, the resulting OD values were measured against the absorbance values of the standard curves, as instructed by the kit manuals.

The supernatant from the initial homogenization of the scraped mucosa samples was diluted (1:50) to reach the working range at 20 to 2000 μg/mL. The total protein content of the diluted scraped mucosal samples was determined using Pierce BCA Protein Assay Kit (#23225, Thermo Fisher Scientific), following the procedure described by Holanda et al. (2020). Absorbance was measured at 562 nm, and protein concentrations (mg/mL) were calculated from a standard curve. These values were then used to normalize the concentrations of TNFα, IL‐6, IL‐8, IgA, IgG, MDA, and protein carbonyl. The MDA concentrations were determined using OxiSelect TBARS MDA Quantitation Assay Kit (#STA330, Cell Biolabs Inc., San Diego, CA), following manufacturer protocol and the method described by Chen et al. (2017). The working range of MDA concentration standard was 0 to 125 μM. The absorbance was read at wavelengths of 532 nm. The OD value was used to calculate the concentration of MDA using the standard curve, described as nmol/mg of protein. The protein carbonyl concentrations were determined using the OxiSelect Protein Carbonyl ELISA Kit (#STA‐310, Cell Biolabs Inc), following the manufacturer protocol and the method described by Cheng et al. (2021). All samples were adjusted to a final concentration of 10 μg/mL. The standard solution covered a working range of 0.375 to 7.500 nmol/mg protein. The absorbance was read at wavelengths of 450 nm. The values of IgA were calculated from the standard curve and expressed as nmol/mg of protein. The concentration of IgA was measured using the pig ELISA kit (#E101–102, Bethyl Laboratories; Montgomery, TX), following the manufacturer protocol and the method previously described by Deng et al. (2022). Mucosa samples were diluted (1:1000) to achieve the proper working range for measurement. The absorbance was read at wavelengths of 450 nm. The values of IgA were calculated from the standard curve and expressed as pg/mg of protein. The IgG concentrations were determined using the pig ELISA kit (#E101–104, Bethyl Laboratories), following manufacturer protocol and the method previously described by Kim et al. (2019). Mucosa samples were diluted (1:3200) to achieve the proper working range for measurement. The absorbance was read at wavelengths of 450 nm. The standard curve was used to calculate the concentration of IgG described as μg/mg of protein. The TNF‐α concentrations were determined using the Porcine TNF‐α DuoSet ELISA Kit (#DY690B, R&D Systems, Minneapolis, MN), following the manufacturer protocol and the method described by Cheng et al. (2022). Absorbance was measured at wavelengths of 450 nm, and the values were calculated from the standard curve and expressed as pg/mg of protein. The concentration of IL‐6 was measured by following instructions of the Porcine IL‐6 DuoSet ELISA Kit (#DY686, R&D Systems), as previously described by Jang and Kim (2019). The absorbance was read at wavelengths of 450 nm. The IL‐6 concentration was quantified from the standard curve and expressed as pg/mg of protein. The concentration of IL‐8 was measured by following instructions of the Porcine IL‐8/CXCL8 DuoSet ELISA Kit (#DY535, R&D Systems) as previously described by Jang and Kim (2019). Mucosa samples were diluted at a 1:20 ratio. The absorbance was read at wavelengths of 450 nm. The IL‐8 concentration was quantified from the standard curve and expressed as pg/mg of protein.

### 2.6 Intestinal Morphology and Crypt Cell Proliferation

**FIGURE 1:**
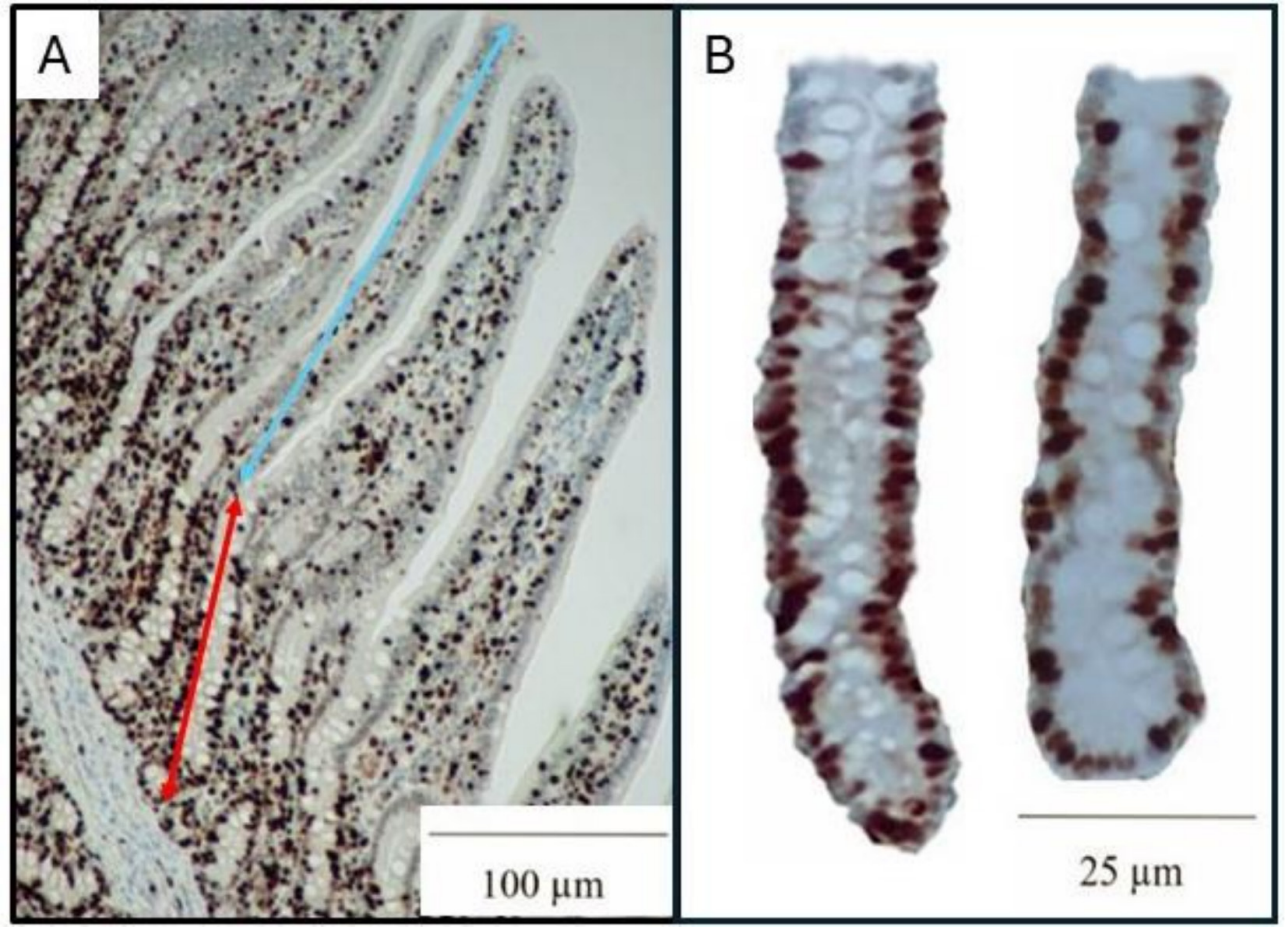
Representative histological images of jejunal tissue used for determination of intestinal morphology and crypt cell proliferation. Mid‐jejunum segments were fixed, processed, and stained with Ki‐67 immunohistochemistry. For each slide, 20 images were taken at 40× magnification of well‐oriented villi and associated crypts (A) to measure villus height (tip of villus to villus–crypt junction, indicated by blue double arrow), crypt depth (villus–crypt junction to base of crypt, indicated by red double arrow). Additional images at 100× magnification of crypts (B) were obtained to quantify the percentage of Ki‐67^+^ cells as an indicator of crypt cell proliferation.

Following collection, mid‐jejunum segments were fixed in 10% formalin for three days. After fixation, the tissues were cut transversely into two pieces, placed in plastic cassettes, and transferred to a 70% ethanol solution for storage. The cassettes were delivered to North Carolina State University College of Veterinary Medicine Histopathology Lab for immunohistochemistry staining with Ki‐67^+^ assay. Processed samples were mounted on slides and evaluated using an Olympus CX31 microscope (Lumenera Corporation; Ottawa, Canada) and the Infinity 2–2 digital CCD software. For each slide, 20 images were taken from various locations, where the entire length of the villus and the associated crypt was easily visible to measure VH, CD, and the villus height to crypt depth (VH:CD) ratio using the Teledyne Lumenera Infinity Analyze 7 software (Lumenera Corporation). Villus height was measured from the tip of the villus to the junction with the crypt, whereas CD was measured from this junction to the base of the crypt (Figure 1). The VH:CD ratio was determined by dividing VH by CD. The lengths of 25 well‐shaped crypts were taken from each slide to calculate the ratio of Ki‐67^+^ cells in a crypt of the mid‐jejunum by operating the software ImageJS. The percentage of Ki‐67^+^ cells was an indicator of the crypt cell proliferation rate (Duarte et al. 2020; Moita et al. 2021).

### 2.7 Statistical Analysis

Data were analyzed using the MIXED procedure (SAS Inc., Cary, NC, USA). Initial BW and sex were considered as blocks. The statistical model included dietary treatment as a fixed effect and blocks as random effects. The LSMEANS procedure was used to calculate mean values. The experimental unit was a pig, individually housed and fed. A power test was performed to determine the required number of replications needed to achieve statistical significance for an anticipated mean difference of 8% at *p* < 0.05. This test used a coefficient of variation of 8%, based on prior studies involving pigs with a similar genetic background conducted at the same research facility (Duarte and Kim 2022a; Duarte and Kim 2024). The power test, assuming a 95% confidence level, indicated that a minimum of 12 replications per treatment was required to achieve 80% power (Aaron and Hays 2004). Statistical differences were considered significant with *p* < 0.05 and tendencies with 0.05 ≤ *p* < 0.10.

## 3 Results

### 3.1 Immune Status and Oxidative Stress Markers

Pigs fed control diet had an MDA concentration of 0.23 nmol/mg, whereas pigs fed diet supplemented with 0.2% mixed macroalgae sulfated polysaccharides had an MDA concentration of 0.29 nmol/mg (Table 2). Protein carbonyl levels were lower (*p* < 0.05) in pigs fed diet supplemented with 0.2% mixed macroalgae sulfated polysaccharides (3.32 nmol/mg) than in pigs fed control diet (4.92 nmol/mg). The IgA concentrations were 2.57 μg/mg in the pigs fed control diet and 2.09 μg/mg in pigs fed diet supplemented with 0.2% mixed macroalgae sulfated polysaccharides, whereas IgG concentrations were 2.22 μg/mg and 1.86 μg/mg, respectively, with no differences between treatments. The TNF‐α concentrations were lower (*p* < 0.05) in pigs fed diet supplemented with 0.2% mixed macroalgae sulfated polysaccharides (0.68 pg/mg) than in pigs fed control diets (0.93 pg/mg). Additionally, IL‐6 concentrations were 10.44 pg/mg in pigs fed control diet and 12.18 pg/mg in pigs fed diet supplemented with 0.2% mixed macroalgae sulfated polysaccharides, showing no differences between treatments. However, IL‐8 concentrations tended to be lower (*p* = 0.064) in pigs fed diet supplemented with 0.2% mixed macroalgae sulfated polysaccharides (0.62 ng/mg) than in pigs fed control diet (0.86 ng/mg).

**TABLE 2:** Intestinal health markers in the jejunal mucosa of pigs fed a diet supplemented with 0.2% of macroalgae sulfated polysaccharides.

Item	Control	Macroalgae sulfated polysaccharides	SEM	*p*
Malondialdehyde, nmol/mg protein	0.23	0.29	0.04	0.250
Protein carbonyl, nmol/mg protein	4.92	3.32	0.46	0.028
Immunoglobulin A, μg/mg protein	2.57	2.09	0.47	0.477
Immunoglobulin G, μg/mg protein	2.22	1.86	0.39	0.525
Tumor necrosis factor‐α, pg/mg protein	0.93	0.68	0.08	0.045
Interleukin‐6, pg/mg protein	10.44	12.18	1.70	0.477
Interleukin‐8, pg/mg protein	0.86	0.62	0.11	0.064

### 3.2 Intestinal Morphology and Crypt Cell Proliferation

Villus height was 561 μm in pigs fed control diet and 561 μm in pigs fed diet supplemented with 0.2% mixed macroalgae sulfated polysaccharides (Table 3). Crypt depth was 283 μm in pigs fed control diet and 277 μm in pigs fed diet supplemented with 0.2% mixed macroalgae sulfated polysaccharides, with no differences between treatments. Similarly, the VH:CD ratio was 1.99 in pigs fed control diet and 1.98 in pigs fed diet supplemented with 0.2% mixed macroalgae sulfated polysaccharides. Crypt cell proliferation, as indicated by Ki‐67^+^ expression, was 27.6% in pigs fed control diet and 26.8% in pigs fed diet supplemented with 0.2% mixed macroalgae sulfated polysaccharides, with no differences observed.

**TABLE 3:** Intestinal morphology and crypt cell proliferation (Ki‐67^+^) in the jejunal mucosa of pigs fed a diet supplemented with 0.2% of macroalgae sulfated polysaccharides.

Item	Control	Macroalgae sulfated polysaccharides	SEM	*p*
Villus height, μm	561	561	18	0.990
Crypt depth, μm	283	277	7	0.569
Villus height to crypt depth ratio	1.99	1.98	0.04	0.828
Ki‐67^+^ ^a^, %	27.6	26.8	0.5	0.215

^a^ Ratio of Ki‐67^+^ to total cells in the crypt.

### 3.3 Growth Performance

Pigs fed control diet had an initial BW of 6.53 kg, whereas pigs fed diet supplemented with 0.2% mixed macroalgae sulfated polysaccharides had an initial BW of 6.52 kg (Table 4), indicating no difference between treatments. On day 7, BW was 7.12 kg in pigs fed control diet and 7.09 kg in pigs fed diet supplemented with 0.2% mixed macroalgae sulfated polysaccharides. By day 21, BW was 12.0 kg in the pigs fed control diet and 11.8 kg in the pigs fed diet supplemented with 0.2% mixed macroalgae sulfated polysaccharides, and by day 35, BW was 20.4 and 20.2 kg, respectively, showing no differences between treatments. The ADG was 84 g in pigs fed control diet and 81 g in pigs fed diet supplemented with 0.2% mixed macroalgae sulfated polysaccharides during phase 1, 351 and 339 g during phase 2, and 599 and 598 g during phase 3, with no effect of dietary treatment on overall ADG (397 g vs. 391 g). Similarly, ADFI was 117 g in pigs fed control diet and 114 g in pigs fed diet supplemented with 0.2% mixed macroalgae sulfated polysaccharides during phase 1, 413 and 451 g during phase 2, and 864 and 886 g during phase 3, with no difference observed in overall ADFI (535 g vs. 557 g). No differences were observed in G:F ratio between treatments, with values of 0.68 and 0.59 during phase 1, 0.84 and 0.76 during phase 2, and 0.70 and 0.68 during phase 3 for pigs fed control diets and pigs fed diet supplemented with 0.2% mixed macroalgae sulfated polysaccharides, resulting in an overall G:F ratio of 0.74 and 0.70, respectively.

**TABLE 4:** Growth performance of pigs fed a diet supplemented with 0.2% of macroalgae sulfated polysaccharides.

Item	Control	Macroalgae sulfated polysaccharides	SEM	*p*
BW, kg				
Day 0	6.53	6.52	0.20	0.962
Day 7	7.12	7.09	0.28	0.880
Day 21	12.0	11.8	0.5	0.768
Day 35	20.4	20.2	0.9	0.872
ADG, g				
Days 0–7 (phase 1)	84	81	11	0.900
Days 7–21 (phase 2)	351	339	28	0.770
Days 21–35 (phase 3)	599	598	35	0.987
Days 0–35 (overall)	397	391	25	0.873
ADFI, g				
Days 0–7 (phase 1)	117	114	13	0.856
Days 7–21 (phase 2)	413	451	33	0.435
Days 21–35 (phase 3)	864	886	49	0.757
Days 0–35 (overall)	535	557	33	0.629
G:F ratio				
Days 0–7 (phase 1)	0.68	0.59	0.10	0.629
Days 7–21 (phase 2)	0.84	0.76	0.03	0.524
Days 21–35 (phase 3)	0.70	0.68	0.02	0.111
Days 0–35 (overall)	0.74	0.70	0.02	0.136

Abbreviations: ADFI, average daily feed intake; ADG, average daily gain; BW, body weight; G:F, gain to feed ratio.

## 4 Discussion

This study aimed to evaluate the impacts of dietary supplementation with a mix of macroalgae sulfated polysaccharides on inflammatory cytokines, immunoglobulins, oxidative stress markers, intestinal morphology, crypt cell proliferation, and growth performance of nursery pigs. The results indicated that the inclusion of 0.2% mixed macroalgae sulfated polysaccharides exerted an anti‐inflammatory response, reducing pro‐inflammatory cytokine expression and oxidative stress in the jejunal mucosa. The jejunum was selected for evaluation because it is the primary site of digestion, nutrient absorption, and immune response in the intestine of pigs (Kim and Duarte 2021; Duarte and Kim 2022b). Consequently, the jejunum provides relevant evidence of how dietary interventions influence intestinal health and performance. This study integrates multiple mechanistic indicators, providing a more detailed understanding of how macroalgae supplementation modulates jejunal physiology in nursery pigs.

Intestinal health refers to the state of homeostasis within the gastrointestinal tract, involving both its structure and function (Pluske et al. 2018). Intestinal health is not always directly associated with pathogens that cause clinical or subclinical illness, morbidity, and mortality in pigs (Kim and Duarte 2021). Intestinal health can also be compromised in the absence of any disease or pathogen. The transition from sow's milk to solid feed, along with environmental and physiological stressors, can lead to decreased feed intake, compromised mucosal immunity, and altered intestinal microbiota, ultimately affecting nutrient absorption and growth (Hu et al. 2013; Jang and Kim 2022; Kick et al. 2012). The immaturity of the digestive and immune systems in nursery pigs further exacerbates these challenges, making dietary interventions that support intestinal health particularly valuable (Zheng et al. 2021).

Weaning stress increases the expression of proinflammatory cytokines in the intestines of nursery pigs, which can lead to a reduction in tight junction proteins and increased intestinal epithelial permeability (Hu et al. 2013; Jang et al. 2024). Xu et al. (2022) reported that pro‐inflammatory cytokines, including IL‐6 and IL‐8, were elevated in pigs at 21 days of age. These cytokines play a key role in recruiting immune cells, further amplifying inflammation and contributing to cell damage (Noh et al. 2015). In this study, the inclusion of 0.2% mixed macroalgae sulfated polysaccharides decrease TNF‐α levels by 27% and tended to decreased IL‐8 by 28% in the jejunal mucosa. This result could be attributed to ulvan and carrageenan, water‐soluble sulfated polysaccharides found in *Ulva* spp. and *Solieria chordalis* (Corino et al. 2021). Macroalgae sulfated polysaccharides exert anti‐inflammatory effects by modulating intracellular signaling pathways, inhibiting key enzymes, and regulating transcription factors (Cian et al. 2018). Specifically, these bioactive compounds, ulvan and carrageenan, have been shown to regulate immune responses by inhibiting key inflammatory pathways, including NF‐κB activation and MAPK signaling cascades (Berri et al. 2017; Flórez‐Fernández et al. 2023). Additionally, macroalgae sulfated polysaccharides can mitigate oxidative stress resulting from inflammation. Inflammation triggers the production of ROS, such as superoxide anion, hydroxyl radicals, and nitric oxide, which lead to oxidative damage and apoptosis (Hussain et al. 2016; Juan et al. 2021; Yang et al. 2007). Macroalgae sulfated polysaccharides can inhibit the expression of inducible nitric oxide synthase and cyclooxygenase‐2, thereby reducing the production of nitric oxide and prostaglandin E2, two key mediators of inflammation (Shen et al. 2024). These sulfated polysaccharides, specifically ulvan and carrageenan, also downregulate TNF‐α, IL‐6, and IL‐1β, which are critical cytokines in the inflammatory cascade, whereas promoting the production of IL‐10, an anti‐inflammatory cytokine, helping to maintain intestinal homeostasis and protect against inflammation‐induced tissue damage (Brito et al. 2016; Chaves et al. 2013; Shen et al. 2024). Consistent with these findings, the inclusion of 0.2% mixed macroalgae sulfated polysaccharides decreased protein carbonyl levels, a marker of oxidative damage to cellular proteins, suggesting that the bioactive compounds exhibit anti‐oxidative effects (Qi et al. 2005; Qi and Sun 2015; Shen et al. 2015). The anti‐oxidative effects of ulvan and carrageenan include scavenging free radicals, metal chelation, and enhancing anti‐oxidative enzyme activity (Yuan et al. 2006). Although lipid oxidation was not affected in this study, mitigation of protein oxidation may contribute to the preservation of intestinal barrier integrity and immune homeostasis in nursery pigs.

Intestinal morphology is essential for nutrient absorption and overall health. However, oxidative stress and inflammation can disrupt this structure, leading to enterocyte apoptosis and impaired nutrient utilization (Duarte et al. 2019). Crypt cell proliferation plays a key role in repairing intestinal epithelium damaged by pathogenic bacteria and their toxins (Duarte and Kim 2024; Sun et al. 2021). In this study, no differences were observed between pigs fed the control diet and those supplemented with macroalgae sulfated polysaccharides for VH:CD ratio or crypt cell proliferation (Ki‐67^+^ cells). The VH:CD ratio, a key indicator of intestinal function, suggests no impact on villus integrity or nutrient absorption (Nabuurs et al. 1993). Likewise, crypt cell proliferation showed minimal variation (27.6% in control vs. 26.8% in supplemented pigs), reinforcing that the primary benefits of macroalgae are likely due to its anti‐inflammatory and anti‐oxidative effects rather than structural modifications of the epithelium.

No differences in growth performance parameters were observed between pigs fed control diet and those supplemented with 0.2% mixed macroalgae sulfated polysaccharides throughout the 35‐day study. This lack of effect may be attributed to the study duration, as potential benefits of macroalgae supplementation on growth might require a longer adaptation feeding period to manifest (Choi et al. 2025). Similar findings have been reported in studies with comparable feeding durations, where no improvements in growth performance were observed. Both McAlpine et al. (2012) and Lee et al. (2019) reported no improvements in growth performance in weaned pigs after 40 and 42 days of supplementation with 300 mg/kg laminarin +240 mg/kg fucoidan and 3.12% *Aurantiochytrium limacinum*, respectively, supporting the idea that relatively short feeding durations may limit observable growth benefits. However, Lee et al. (2019) did observe a 30% reduction in cortisol levels, suggesting that macroalgae supplementation may influence stress and immune responses even in the absence of growth effects. These findings align with evidence that macroalgae can promote beneficial intestinal microbiota and exert anti‐inflammatory and anti‐oxidative effects, even without short‐term improvements in growth performance (Berri et al. 2017; Cheng et al. 2021; Flórez‐Fernández et al. 2023). Therefore, improvements in intestinal health and immune function may precede, and potentially enable, later growth responses, which may only become evident with longer feeding durations.

Although polysaccharide composition on macroalgae was not evaluated in this study, it is well established that cell wall polysaccharides are the major components of macroalgae, accounting for 38% to 54% of their dry weight, and they could offer potential benefits to intestinal health (Flórez‐Fernández et al. 2023). Polysaccharides are among the most abundant compounds in macroalgae and have been shown to exhibit a wide variety of biological and pharmacological activities (Guo et al. 2020; Walsh et al. 2013). However, detailed structural characterization of the sulfated polysaccharides (molecular weight distribution and sulfation patterns) was beyond the scope of this study, and the consistent functional responses observed highlight the biological relevance of the mixed macroalgae sulfated polysaccharides. Future research including chemical characterization will be valuable to further define the specific compounds and mechanisms responsible for these effects.

This study demonstrates that a diet supplemented with 0.2% mixed macroalgae sulfated polysaccharides reduced inflammation and protein oxidation in the jejunum of nursery pigs, highlighting its potential role in supporting intestinal health. Although lipid oxidation and growth performance were not affected during the nursery phase, the reduction in protein carbonyl suggests partial mitigation of oxidative stress and potential long‐term health benefits. Given these findings, future research should evaluate additional oxidative stress markers and investigate the long‐term effects of macroalgae on intestinal health, disease resistance, and overall pig performance, particularly under challenge conditions.

## Conflicts of Interest

M.G.S. and M.A.R. are affiliated with Olmix S. A. The other authors (Y‐C.C., Y.R.G‐D., and S.W.K) declare no conflicts of interest.
